# Geographical variation and associated factors of childhood measles vaccination in Ethiopia: a spatial and multilevel analysis

**DOI:** 10.1186/s12889-019-7529-z

**Published:** 2019-08-30

**Authors:** Tesfahun Taddege Geremew, Lemma Derseh Gezie, Ayenew Negesse Abejie

**Affiliations:** 1 Ethiopian Field Epidemiology and Laboratory Training Program, Department of Epidemiology and Biostatistics, Institute of Public Health, College of Medicine and Health Sciences, University of Gondar, P.O. Box 169, Gondar, Ethiopia; 2 Department of Reproductive Health and Population Studies, School of Public Health, College of Medicine and Health Sciences, Bahir Dar University, P.O. Box 79, Bahir Dar, Ethiopia; 3 Department of Epidemiology and Biostatistics, Institute of Public Health, College of Medicine and Health Sciences, University of Gondar, P.O. Box 169, Gondar, Ethiopia; 4 Department of Human Nutrition and Food Sciences, College of Health Sciences, Debre Markos University, P.O. Box 269, Debre Markos, Ethiopia; 5 Academic Center of Excellence for Human Nutrition, Food Science and Post-harvest Technology, Hawassa University, Hawassa, Ethiopia

**Keywords:** Measles, Vaccination, Spatial, Multilevel, Ethiopia

## Abstract

**Background:**

In Ethiopia, despite considerable improvement of measles vaccination, measles outbreaks is occurring in most parts of the country. Understanding the neighborhood variation in childhood measles vaccination is crucial for evidence-based decision-making. However, the spatial pattern of measles-containing vaccine (MCV1) and its predictors are poorly understood. Hence, this study aimed to explore the spatial pattern and associated factors of childhood MCV1 coverage.

**Methods:**

An in-depth analysis of the 2016 Ethiopia demographic and health survey data was conducted, and a total of 3722 children nested in 611 enumeration areas were included in the analysis. Global Moran’s I statistic and Poisson-based purely spatial scan statistics were employed to explore spatial patterns and detect spatial clusters of childhood MCV1, respectively. Multilevel logistic regression models were fitted to identify factors associated with childhood MCV1.

**Results:**

Spatial hetrogeniety of childhood MCV1 was observed (Global Moran’s I = 0.13, *p*-value < 0.0001), and seven significant SaTScan clusters of areas with low MCV1 coverage were detected. The most likely primary SaTScan cluster was detected in the Afar Region, secondary cluster in Somali Region, and tertiary cluster in Gambella Region. In the final model of the multilevel analysis, individual and community level factors accounted for 82% of the variance in the odds of MCV1 vaccination. Child age (AOR = 1.53; 95%CI: 1.25–1.88), pentavalent vaccination first dose (AOR = 9.09; 95%CI: 6.86–12.03) and third dose (AOR = 7.12; 95%CI: 5.51–9.18, secondary and above maternal education (AOR = 1.62; 95%CI: 1.03–2.55) and media exposure were the factors that increased the odds of MCV1 vaccination at the individual level. Children with older maternal age had lower odds of receiving MCV1. Living in Afar, Oromia, Somali, Gambella and Harari regions were factors associated with lower odds of MCV1 from the community-level factors. Children far from health facilities had higher odds of receiving MCV1 (AOR = 1.31, 95%CI = 1.12–1.61).

**Conclusion:**

A clustered pattern of areas with low childhood MCV1 coverage was observed in Ethiopia. Both individual and community level factors were significant predictors of childhood MCV1. Hence, it is good to give priority for the areas with low childhood MCV1 coverage, and to consider the identified factors for vaccination interventions.

## Background

Measles is a very contagious respiratory disease caused by measles virus and spreads through respiratory droplets when an infected person coughs or sneezes [1]. It is a vaccine preventable disease that can cause serious illness, lifelong complications and death. In 2014, it was estimated that 114,900 deaths due to measles had occurred globally, of which 73,914 deaths (93%) were occurred in Africa [2, 3]. Before starting measles vaccination program, nearly 90% of children aged under-15 years were infected with measles [4, 5]. In the era of expanded immunization program, the global measles deaths declined by three-fourth from 2000 to 2014 [2, 6, 7], but measles is still considered as a public health emergency that requires immediate notification and rapid public health response [5]. World Health Organization (WHO) has targeted a global elimination of measles to reduce annual incidence rates (IRs) to less than five cases per million population, which requires more than 90% coverage of at least one dose of Measles-Containing Vaccine (MCV1) by the end of 2015 and more than 95% coverage by 2020 in all countries [5]. In 2015, MCV1 coverage had reached 85% globally, and the measles deaths declined by 79% as compared to 2000 [4]. However, the 2015 measles vaccination goal was not met and measles IR remained relatively unchanged between 2013 and 2014 [2].

Ethiopia accounted for 3.4% of the estimated 20.8 million infants globally who did not receive MCV1 through routine immunization services in 2015 [8], and 9% of the global measles mortality is attributed to Ethiopia [6, 9]. In Ethiopia, childhood immunization coverage is improved through the combine effect of Reaching Every District (RED) approach, health extension program and implementation of Enhanced Routine Immunization Activities (ERIAs) [7, 10–12]. It is expected that measles immunization coverage should have an inverse relation with the of the incidence rate of measles [13]. However, despite a considerable improvement of childhood MCV1 in Ethiopia, measles outbreaks are occurring continuously in most parts of the country. As the MCV1 coverage increased from 59% in 2005 to 84% in 2014 [4, 7, 9, 11, 14], the incidence rate of confirmed measles cases per 100,000 population increased from 0.6 in 2005 to 11.2 in 2014 [11]. This continuous occurrence of measles outbreak irrespective of measles vaccination could be attributed to spatial heterogeneity of measles vaccination [5, 11].

Spatial heterogeneity of measles vaccination coverage can delay measles elimination even in countries with high average nationwide vaccination coverages [15, 16].

Understanding the neighborhood variation in measles vaccination is crucial for evidence-based decision-making in meales perevention and control program and detecting spatial heterogeneity is useful to identify gaps in the performance of measles immunization programme that could not be identified through the routine monitoring of vaccination coverage [5]. However, studies are limited on the spatial pattern of childhood MCV1 and its associated factors in Ethiopia. Hence, this study aimed to explore the spatial pattern of MCV1 and associated factors among children.

## Methods

### Study settings

The Ethiopia demographic and health survey (EDHS) is a national and subnational representative household survey, which is conducted every 5 year. Ethiopia is second most populous country in Africa, located in the horn of Africa. Administratively, Ethiopia is sub-divided into 11 geographical regions; each region is again sub-divided into zones, and zones into districts. The districts in-further are sub-divided into kebeles (the smallest administrative units). During the 2007 Ethiopian population and housing census, each kebele was sub-divided into enumeration areas (EAs), which were used as a sampling frame for the 2016 EDHS.

The health system of Ethiopia, which focuses on a preventive health care, is organized into three-health tire system: Primary health care unit (PHCU), general hospital and specialized hospital. Routine child immunization is primarily provided at PHCUs through static and outreach sites [17], and the availability of routine childhood vaccination services including measles vaccine in Ethiopia is 80% [18].

### Study population and eligibility

Children aged 12–35 months living in the selected EAs were the study population. All children aged 12–35 months were included and children whose geographical locations were not available at the global positioning system (GPS) were excluded from this study. Hence, a total of 23 EAs, which consisted 133 children, were excluded from the analysis.

### Sampling technique and sample size

The 2016 EDHS used a stratified, two-stage cluster sampling design using the enumeration areas (EAs) of the 2007 Ethiopian population and housing census (PHC) as the primary sampling unit (PSU) and households as the secondary sampling unit (SSU). In the first stage, 645 EAs (202 in urban areas and 443 in rural areas) were selected with probability proportional to EAs size (PPS) from the complete list of 84,915 EAs created for the 2007 PHC sampling frame. In the second stage of selection, a fixed number of 28 households per cluster were selected with an equal probability systematic selection from the newly created household listing. Finally, 16,650 households were successfully interviewed, yielding a response rate of 98%. A total of 3722 children aged 12–35 months nested in 611 EAs were included in this analysis. The detailed methodology has been published in the 2016 EDHS final report [19].

### Data source and extraction

The 2016 Ethiopia demographic and health survey datasets, which are publicly available to all registered users, were requested and downloaded from Measure DHS program website (https://www.dhsprogram.com/data/dataset_admin). Childhood MCV1 status and its potential predictor variables at individual and community level were extracted accordingly.

### Study variables

#### Dependent variables

MCV1 vaccination status of the last child aged 12–35 months was the outcome variable of this study. If the child had received MCV1, it was classified as “Yes”, otherwise “No”, and the data was collected from written vaccination records (including the infant immunization card and other health cards), and mothers’ verbal reports.

#### Independent variables

Both individual and community level characteristics were considered as potential the potential predictor variables for MCV1. Table 1 depicted the potential predictor variables included in this study along with their categories. Individual-level (level I) variables include socio-demographic and economic characteristics, while community-level (level II) variables include the common characteristics of study subjects in an enumeration area such as region and place of residence.

**Table 1:** Independent variables and categorization

Variables	Categories
1. Individual level factors (level I)	
Child characteristics	
Age of child (months)	(1) 12–23; (2) 24–35
Sex of the child	(1) Male; (2) Female
Birth order	(1) 1; (2) 2–3; (3) 4–5; (4) 6+
Acceptance of the child	(1) Wanted then, (2) Wanted later & (3) Wanted no more
DPT1-HepB1-Hib1	(0) Not vaccinated; (1) Vaccinated
DPT3-HepB3-Hib3	0) Not vaccinated; (1) Vaccinated
Maternal/paternal characteristics	
Mother’s age (years)	(1)15–19; (2) 20–34; (2) 35–49
Religion	(1)Orthodox; (2)Muslim; (3)Protestant; (4) Others
Mother’s education	(1) No education; (2)Primary; (3) Secondary and higher
Father’s education	1) No education; (2)Primary; (3) Secondary and higher
Wealth index	(1) Poorest; (2)Poorer; (3) Rich; (4) Richer; (5)Richest
Sex of household head	(1) Male; (2) Female
Number of living children	(1) 1; (2) 2–3; (3) 4–5; (4) 6+
Head of household	(1) Male; (2) Female
Mother’s relation to the head of the household	(1) Wife; (2) head; (3)Daughter; (4) Others
Regular media exposure	(0) No; (1) Yes
2. Community level factors (Level II)	
Residence	(1) Urban; (2) Rural
Region	(1) Addis Ababa; (2)Tigray; (3) Afar; (4) Amhara; (5) Oromiya; (6) Somali; (7) Benishangul-Gumuz; (8) SNNPR; (9) Gambella; (10) Harari; (11) Dire Dawa
Distance to health facility	(1) A big problem, (2) Not a big problem

### Operational definitions

Clustering: An unusual aggregation of children with the same MCV1 status (vaccinated or unvaccinated) in a specific geographical area [20, 21].

Measles-containing vaccines (MCV): Vaccines containing antigens of measles only (M) or a combination of measles with rubella (MR), mumps (MM, MMR) and varicella (MMRV) vaccines [22].

Children who received MCV1: Children who received a one dose measles-containing vaccine for the first time at any time before the survey (based on the evidence from vaccination cards, health facility records, or the mother’s report) [19].

Exposure to mass media: Women were asked how often they read a newspaper, listened to the radio, or watched television per week. Those who responded at least once a week are considered to be regularly exposed to that form of media [19].

### Data management and statistical analysis

We re-categorized children’s age, mother’s age, birth order, mother’s educational status, religion, number of under-five children, and mother’s relation to the head of the household to better suit with other studies for comparison. We used SaTScan 9.6 (https://www.satscan.org/) [23] and ArcGIS version 10.3 (http://www.esri.com/) for spatial analysis, and Stata version 14 (https://www.stata.com/) [24] for non-spatial statistical analysis.

Sample weights were applied to compensate for the unequal probability of selection between the strata that has been geographically defined. The detailed explanation of the weighting procedure can be found in the methodology of the EDHS final report [19].

Spatial Analysis: The aggregated MCV1 count data was joined to the geographic coordinates based on each EA unique identification code. To evaluate whether the pattern expressed is clustered, dispersed, or random across the study areas, global spatial autocorrelation was assessed with ArcGIS using the Global Moran’s I statistic (Moran’s I) [25]. When *p*-value indicates statistical significance, a positive Moran’s I index value indicates tendency toward clustering while a negative Moran’s I index value indicates tendency toward dispersion [25].

In the presence of positive global spatial autocorrelation, we employed Kulldorff’s method of purely spatial scan statistic using the discrete Poisson probability model in SaTScan to detect the local spatial clusters of areas with high or low childhood MCV1 coverages. “SaTScan™ is a trademark of Martin Kulldorff, and a software which was developed under the joint auspices of (i) Martin Kulldorff, (ii) the National Cancer Institute, and (iii) Farzad Mostashari of the New York City Department of Health and Mental Hygiene” [21, 23, 26]. SaTScan uses a circular window that moves systematically throughout the study area to identify statistically significant SaTScan clustering of areas with the same childhood MCV1 coverage. We used a maximum of 10% (to avoid the detection of large clusters) of the population at risk for the spatial cluster size and the analysis was done using standard Monte Carlo hypothesis testing with 999 Monte Carlo replicates. A cluster is statistically significant when its log likelihood ratio (LLR) is greater than the Standard Monte Carlo critical value (C.V) for 0.05 significance level or *p*-value < 0.05 [21, 23].

Multilevel logistic regression Analysis: The data of this analysis included 3722 children nested within 611 EAs. Hence, considering the hierarchical nature of the data, multilevel logistic regression models were fitted to identify community and individual level factors associated with childhood MCV1. Four models were fitted: the first model without any explanatory variable (empty model) to detect the existence of possible contextual effect (model I), the second model with individual-level variables (model II), the third model with community-level variables (Model III) and the fourth model (Model IV) with both the individual- and community-level variables. Model comparison was done using deviance information criteria (DIC) and Akaike’s Information Criterion (AIC). Finally, the fourth model (model IV) with the smallest value of the information criterion was selected as the final best fit model.

For measures of association (fixed effect), adjusted odds ratio with 95% confidence intervals was used to declare statistical significance.

For measures of variation (random effects), Intra-class correlation coefficient (ICC), median odds ratio (MOR) and proportional change in variance (PCV) statistics were computed. ICC is a measure of within-cluster variation, the variation between individuals within the same cluster, and it was calculated using the formula: $\mathrm{ICC}=\frac{V_A}{V_A+\frac{\uppi^2}{3}}=\frac{V_A}{V_A+3.29}$, where V_A_ is the estimated variance in each model, which has been described elsewhere [27].

The total variation attributed to individual or/and community level factors at each model was measured by the proportional change in variance (PCV), which was calculated as $\mathrm{PCV}=\frac{{\mathrm{V}}_A-{\mathrm{V}}_{\mathrm{B}}}{{\mathrm{V}}_{\mathrm{A}}}$, where V_A_ = variance of the initial model, and V_B_ = variance of the model with more terms [27].

The MOR is the median odds ratio between the individual of higher propensity and the individual of lower propensity when comparing two individuals from two different randomly chosen clusters and it measures the unexplained cluster heterogeneity, the variation between clusters by comparing two persons from two randomly chosen different clusters. It was computed using the formula: $\mathrm{MOR}=\exp \left(\sqrt{2\ \mathrm{x}\ {\mathrm{V}}_{\mathrm{A}}}\ \mathrm{x}\ 0.6745\right)\approx \exp \left(0.95\sqrt{{\mathrm{V}}_{\mathrm{A}}}\right)$, where V_A_ is the cluster level variance [27, 28]. The MOR measure is always greater than or equal to 1. If the MOR is 1, there is no variation between clusters [29].

### Ethical considerations

The data of the 2016 Ethiopia demographic and health survey was used for this study with a permission from the Measure DHS program after being registered and submiting a request with briefly stated objctives of the study. Ethical approval was obtained from the Institutional Review Board of the Institute of Public Health of the University of Gondar and the ICF International Institutional Review Board. The data has been used only for this registered research and it could not be passed on to other researchers. The shape files for Ethiopia’s administrative boundaries were obtained from the openAFRICA website [https://africaopendata.org/dataset/ethiopia-shapefiles] [30]. The detaile of the ethical issues has been published in the 2016 EDHS final report, which can be accessed at: http://www.dhsprogram.com/publications [19].

## Results

### Characteristics of study participants and childhood MCV1 *prevalence*

Nearly half of the children (51%) were in the 12–23 months age group with the mean age of 23 months (± 7SD). Nearly 70% of the children had received pentavalent first dose vaccine (DPT1-HepB1-Hib1) and 49% of children had received third dose of pentavalent vaccine (DPT3-HepB3-Hib3). Over 63% of mothers of the children had no education, one-fourth of them were in the poorest wealth quintile, and 81% had no regular media exposure.

The overall prevalence of childhood MCV1 in Ethiopia was 54.3% (95% CI = 52.7–55.9). Relatively the highest MCV1 prevalence (94%) was in Addis Ababa town, and lowest in Afar region (29%). The distance to a health facility was a big problem in the three-fifth (60%) of respondents (Table 2).

**Table 2:** Percentage of children age 12–35 months who received MCV1 at any time before the survey (weighted) by background characteristics, Ethiopia, 2016

Background Characteristics	Total number of Children		Children who received MCV1	
	N	%	N	%
Individual level characteristics				
Child Age (in Months) (Mean = 22.95 (SD ± 7.04)				
12–23	1976	51.0	1069	54.1
24–35	1901	49.0	1036	54.5
Sex of child				
Male	1935	49.9	1056	54.6
Female	1941	50.1	1049	54
Birth Order				
1	753	19.4	451	59.9
2–3	1161	29.9	674	58.1
4–5	900	23.2	475	52.8
6+	1063	27.4	505	47.5
Wanted child				
Wanted then	2832	73.1	1491	52.7
Wanted later	702	18.1	430	61.3
Wanted no more	343	8.8	183	53.5
DPT1-HepB1-Hib1 vaccine				
Not vaccinated	1158	29.9	121	10.4
Vaccinated	2718	70.1	1984	73
DPT3-HepB3-Hib3 vaccine				
Not vaccinated	1983	51.2	505	25.5
Vaccinated	1893	48.8	1599	84.5
Maternal age (years)				
15–19	137	3.5	77	56.2
20–34	2850	73.5	1587	55.7
35–49	889	22.9	441	49.6
Religion				
Orthodox	1349	34.8	887	65.8
Muslim	1530	39.5	617	40.4
Protestant	861	22.2	529	61.4
Others^a^	136	3.5	71	52.2
Mother’s educational				
No education	2474	63.8	1188	48
Primary	1100	28.4	669	60.8
Secondary & Higher	302	7.8	248	82.1
Husband/partner’s education (*N* = 3655)				
No education	1746	47.8	820	47
Primary	1462	40.0	838	57.3
Secondary & Higher	447	12.2	319	71.4
Mother’s occupation				
Did not work	2071	53.4	1024	49.4
Non-Professional	1731	44.7	1025	59.2
Professional	74	1.9	56	75.7
Father’s occupation (*N* = 3655)				
Did not work	275	7.5	111	40.2
Nonprofessional	620	17.0	303	48.9
Professional	2760	75.5	1564	56.7
Maternal relation to the household dead				
Wife	3091	79.7	1670	54
Head	406	10.5	224	55.2
Daughter	211	5.4	122	57.8
Others^b^	169	4.4	89	52.7
Sex of the household head				
Male	3314	85.5	1792	54.1
Female	562	14.5	312	55.5
Number of living children				0
1	684	17.6	413	60.4
2–3	1281	33.0	759	59.3
4–5	1022	26.4	529	51.8
6+	890	23.0	404	45.4
Maternal health care decision making (*N* = 3655)				
Jointly with her husband	2439	66.7	1333	54.7
Husband/partner alone	727	19.9	351	48.3
By herself alone	479	13.1	286	59.6
Others	10	0.3	8	80
Wealth index				
Poorest	965	24.9	412	42.7
Poorer	860	22.2	445	51.7
Middle	808	20.8	419	51.9
Richer	676	17.4	393	58.1
Richest	567	14.6	436	76.9
Mass Media Exposure				
Not regular	3154	81.4	1601	50.8
Regular	722	18.6	504	69.7
Community level characteristics				
Region				
Addis Ababa	95	2.5	90	93.8
Tigray	279	7.2	227	81.1
Afar	42	1.1	12	28.6
Amhara	718	18.5	434	60.4
Oromia	1712	44.2	722	42.1
Somali	132	3.4	47	35.6
Benishangul Gumuz	43	1.1	32	76.2
SNNPR	818	21.1	517	63.1
Gambella	10	0.2	6	60
Harari	10	0.2	5	50
Dire Dawa	17	0.4	14	82.4
Residence				
Urban	433	11.2	337	77.8
Rural	3443	88.8	1768	51.4
Distance to health facility				
Big Problem	2332	60.2	1114	47.8
Not a big problem	1545	39.8	991	64.1
Total	3876	100	2105	54.3

^a^Catholic, traditional and other unclassified

^b^Daughter-in-low, sister, other relative, no relation

### Spatial pattern of childhood MCV1

**Fig. 1:**
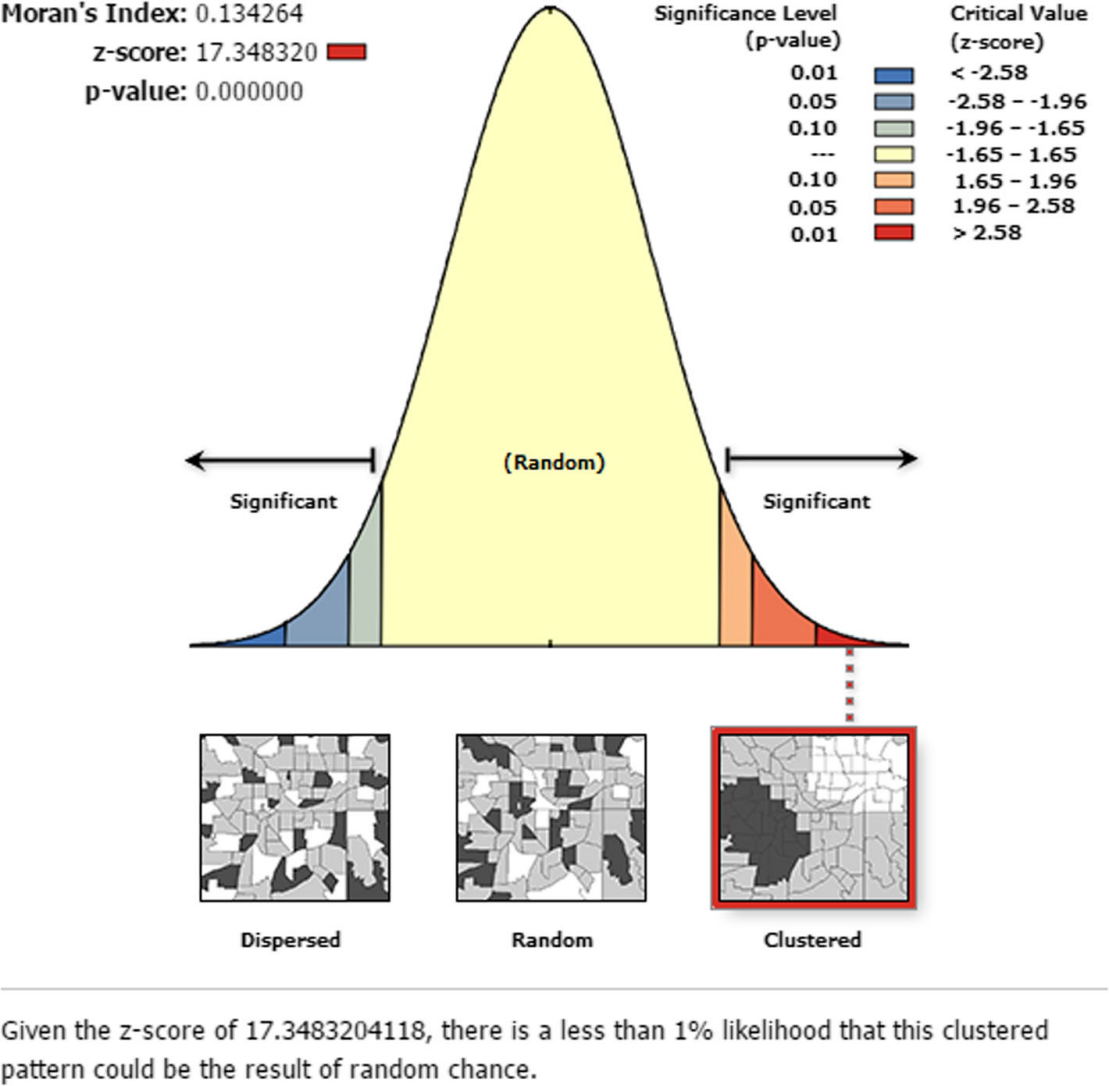
The global spatial autocorrelation based on feature locations and attribute values (MCV1) across the study areas in Ethiopia, 2016

The global spatial autocorrelation analysis based on feature locations and attribute values revealed a clustering pattern of childhood MCV1 across the study areas (Global Moran’s I = 0.134, *p*-value < 0.0001) (Fig. 1).

The SaTScan spatial analysis detected a total of seven statistically significant SaTScan clusters areas with low childhood MCV1 coverage, which means that the prevalence of MCV1 is lower inside the SaTScan circular window compared to outside the SaTScan window. The most likely primary SaTScan cluster of areas with low coverage of MCV1 was detected in Afar region (LLR = 33.79, *p* < 0.01), specifically in Zone 1, Zone 2 and Zone 4 administrative zones and the most likely secondary Spatial SaTScan cluster (LLR = 26.76, *p* < 0.01) in Eastern Ethiopia, specifically in Afder, Shabelle, Korah, Doolo, Nogob, Jarar, Fatan administrative zones of Somali region.

**Fig. 2:**
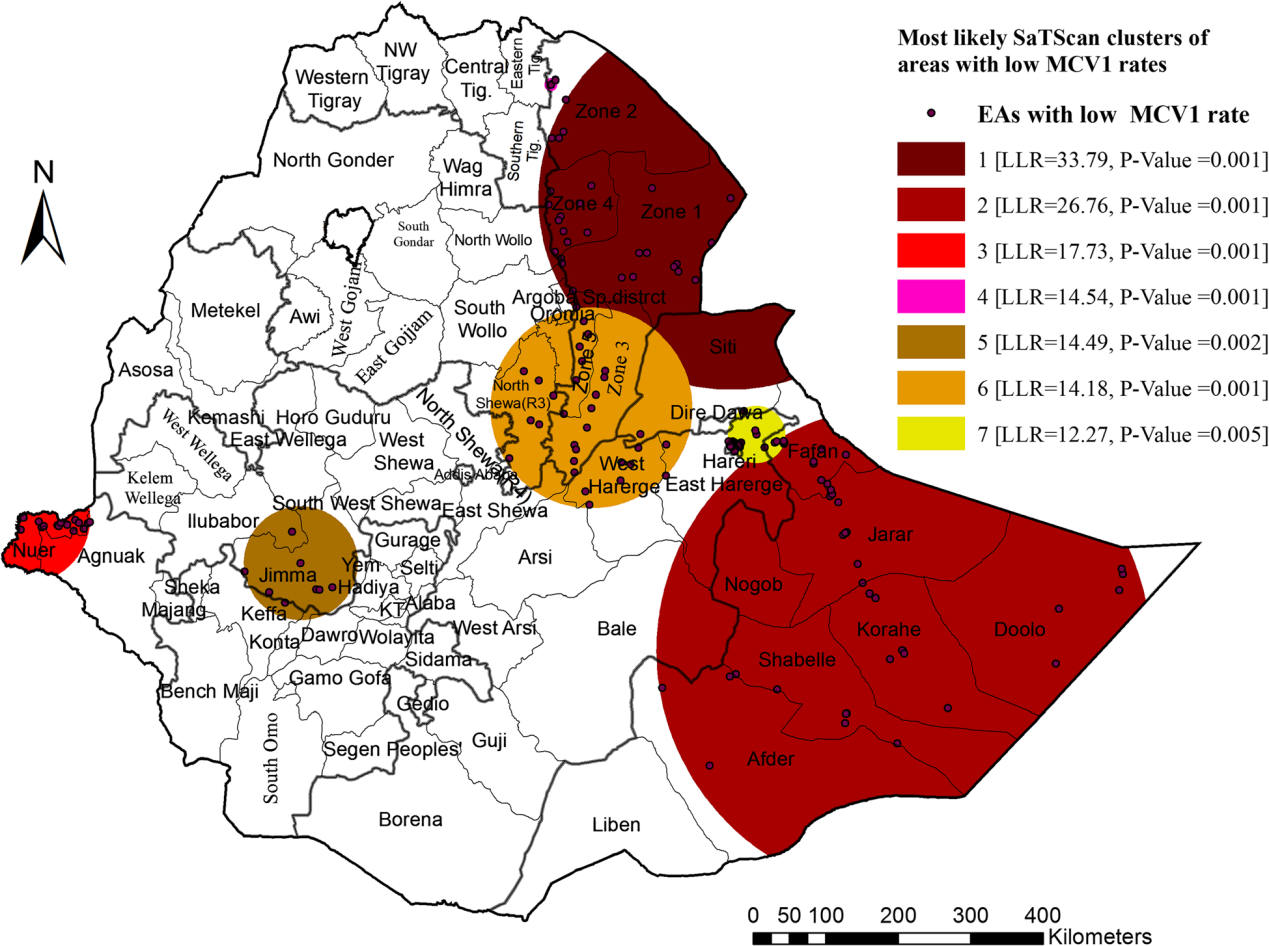
The spatial clustering of areas with low childhood MCV1 coverage in Ethiopia, 2016

The 3rd and 4th most likely clusters of areas with low rates of MCV1 were detected in Nuer and Agnuak Administrative zones of Gambella region (LLR = 17.73, *P* < 0.01) and Zone 2 administration of Afar region (LLR = 14.54, *P* < 0.01), respectively. In addition, the 5th most likely SaTScan cluster (LLR = 14.49, *P* < 0.01) was detected in Illuababur and Jimma Administrative zones of Oromia Region, while the 6th most likely SaTScan cluster (LLR = 14.18, *P* < 0.01) in Zone 3 and Zone 4 of Afar region, North Shewa of Amhara region, West and East Hararge of Oromia Region and Siti zone of Somali region. The 7th most likely SaTScan cluster (LLR = 12.27, *P* < 0.01) was detected in East Hararge Zone of Oromia region, Harari Region and Fagan Zone of Somali region (Table 3 and Fig. 2).

**Table 3:** The most likely clusters from a purely spatial scan statistic (discrete Poisson model) of children received MCV1 in Ethiopia, 2016

Clusters	Regional location (Zones)	Number of clusters	Radius (km)	LLR^a^	C.V^b^	P-Value
Primary cluster	Afar Region (Zone 1, Zone 2 & Zone 3)	30	265.1	33.79	9.85	0.001
Secondary cluster	Somali Region (Afder, Shabelle, Korah, Doolo, Nogob, Jarar, Fatan)	42	339.6	26.76	9.83	0.001
3rd cluster	Gambella Region (Nuer & Agnuak)	15	91.7	17.73	9.45	0.001
4th cluster	Afar Region (Zone 2)	2	8.9	14.54	9.28	0.001
5th cluster	Oromia Region (Illuababur & Jimma)	8	78.7	14.49	9.45	0.002
6th cluster	Afar Region (Zone 3 and 4), Amhara Region (North Shewa), Oromia Region (West and East Hararge) and Somali Region (Siti)	28	139.1	14.18	9.40	0.001
7th cluster	Oromia Region (East Hararge Zone), Harari and Somali Region (Fatan Zone)	26	39.8	12.27	9.69	0.005

^a^Log likelihood ratio

^b^Standard Monte Carlo Critical Value for 0.05 significance level

### Factors associated with the childhood MCV1

In the multilevel analysis, both individual and community level variables were included (Table 4), and the final best fit model (model IV) revealed that child’s age; first and third dose pentavalent vaccination status; maternal age, maternal education; low household wealth index; region; and distance from health facility were factors significantly associated with childhood first dose measles vaccination.

**Table 4:** Multilevel logistic regression analysis of individual and community level factors associated with childhood MCV1 in Ethiopia, 2016

Variables	Model II AOR (95% CI)	Model III AOR (95% CI)	Model IV AOR (95% CI)
Individual level factors			
Child characteristics			
Child’s age (months)			
12–23	1.00	–	1.00
24–35	1.53 (1.2–1.88)	–	1.53 (1.25–1.88)
Sex of child			
Male	1.00	–	1.00
Female	1.11 (0.92–1.35)	–	1.10 (0.91–1.34)
Birth Order			
1	1.00	–	1.00
2–3	1.01 (0.62–1.63)	–	1.00 (0.61–1.63)
4–5	1.03 (0.55–1.93)	–	1.03 (0.55–1.92)
6+	0.86 (0.41–1.81)	–	0.87 (0.42–1.83)
Child Wanted			
Wanted then	1.00	–	1.00
Wanted later	0.93 (0.70–1.23)	–	0.94 (0.71–1.25)
Wanted no more	1.52 (0.98–2.37)	–	1.58 (1.00–2.47)
DPT1-HepB1-Hib1 (Pentavalent first dose) vaccine			
Not vaccinated	1.00	–	1.00
Vaccinated	9.23 (6.98–12.22)	–	9.09 (6.86–12.03)
DPT3-HepB3-Hib3 (Pentavalent third dose) vaccine			
Not vaccinated	1.00	–	1.00
Vaccinated	8.15 (6.33–10.48)	–	7.12 (5.51–9.18)
Maternal Characteristics			
Maternal age (in years)			
15–19	1.00	–	1.00
20–34	0.46 (0.28–0.78)	–	0.45 (0.27–0.76)
35–49	0.49 (0.27–0.90)	–	0.45 (0.25–0.82)
Religion			
Orthodox Christian	1.00	–	1.00
Muslim	0.71 (0.54–0.94)	–	0.95 (0.65–1.38)
Protestant	1.04 (0.74–1.46)	–	1.32 (0.85–2.03)
Others^a^	1.05 (0.54–2.03)	–	1.32 (0.66–2.62)
Maternal Education			
No education	1.00	–	1.00
Primary	1.00 (0.78–1.29)	–	0.99 (0.77–1.28)
Secondary and higher	1.71 (1.10–2.66)	–	1.62 (1.03–2.55)
Mother’s occupation			
Did not work	1.00	–	1.00
Non-professional	1.07 (0.86–1.33)	–	1.04 (0.83–1.29)
Professional	0.97 (0.40–2.35)	–	0.93 (0.38–2.25)
Mather’s relation to the household head			
Wife	1.00	–	1.00
Head	0.72 (0.37–1.42)	–	0.70 (0.35–1.38)
Daughter	1.39 (0.83–2.32)	–	1.35 (0.81–2.26)
Others^b^	1.08 (0.61–1.91)	–	1.05 (0.58–1.87)
Sex of the household head			
Male	1.00	–	1.00
Female	1.54 (0.83–2.87)	–	1.57 (0.84–2.93)
Number of living children	1.00		
1	1.00	–	1.00
2–3	1.21 (0.74–1.97)	–	1.21 (0.74–1.98)
4–5	1.26 (0.66–2.40)	–	1.29 (0.68–2.47)
6+	1.16 (0.55–2.48)	–	1.18 (0.55–2.53)
Wealth index			
Poorest	1.00	–	1.00
Poorer	1.12 (0.83–1.52)	–	1.16 (0.85–1.59)
Middle	0.94 (0.68–1.30)	–	0.99 (0.71–1.40)
Richer	1.06 (0.75–1.50)	–	1.18 (0.83–1.70)
Richest	1.35 (0.93–1.98)	–	1.34 (0.82–2.20)
Regular exposure to mass media			
No	1.00	–	1.00
Yes	1.33 (0.98–1.80)	–	1.32 (0.97–1.79)
Community level factors			
Region			
Addis Ababa	–	1.00	1.00
Tigray	–	0.72 (0.30–1.73)	0.65 (0.27–1.56)
Afar	–	0.04 (0.02–0.09)	0.32 (0.13–0.81)
Amhara	–	0.25 (0.10–0.58)	0.46 (0.19–1.11)
Oromia	–	0.12 (0.05–0.27)	0.24 (0.10–0.58)
Somali	–	0.05 (0.02–0.13)	0.39 (0.16–0.95)
Benishangul	–	0.60 (0.25–1.47)	0.63 (0.25–1.56)
SNNPR	–	0.32 (0.14–0.75)	0.42 (0.17–1.01)
Gambella	–	0.15 (0.06–0.36)	0.39 (0.16–0.99)
Harari	–	0.11 (0.04–0.25)	0.27 (0.11–0.66)
Dire Dawa	–	0.63 (0.25–1.60)	0.69 (0.27–1.79)
Residence			
Urban	–	1.00	1.00
Rural	–	0.30 (0.21–0.44)	1.03 (0.64–1.65)
Distance to Health facility			
Big problem	–	1.00	1.00
Not a big problem	–	1.42 (1.17–1.72)	1.31 (1.12–1.61)

^a^Catholic, traditional and other unclassified

^b^Daughter-in-low, sister, other relative, no relation

Individual level factors: Children aged 24–35 months old were 1.53 times (AOR = 1.53; 95% CI: 1.25–1.88) more likely to be vaccinated for MCV1 than children aged 12–23 months old. Children who have received first dose pentavalent vaccine (DPT1-HepB1-Hib1) (AOR = 9.09; 95% CI: 6.86–12.03) and third dose pentavalent vaccine (DPT3-HepB3-Hib3) (AOR = 7.12; 95% CI: 5.51–9.18) were more likely to receive first dose of measles vaccine. Children whose mothers’ age 20–34 years were 55% (AOR = 0.45; 95% CI: 0.27–0.76) less likely to receive MCV1 compared to those children whose mothers age 15–19 years. In addition, children with maternal age 35–45 years were 55% (AOR = 0.45; 95% CI: 0.25–0.82) less likely to receive MCV1 compared to those children whose mothers age 15–19 years. Children with secondary and higher maternal education were more likely to receive MVC1 vaccination (AOR = 1.62; 95% CI: 1.03–2.55) compared to children whose mothers had no education (Table 4).

Community level factors: Children in Afar (AOR =0.32; 95% CI: 0.13–0.81), Oromia (AOR 0.24; 95% CI 0.10–0.58), Somali (AOR =0.39; 95% CI: 0.16–0.95), Gambella (AOR = 0.39; 95%CI: 0.16–0.99) and Harari (AOR = 0.27; 95%CI: 0.11–0.66) regions were less likely to receive MCV1 vaccination compared to children in Addis Ababa. The odds of MVC1 vaccination were increased by 31% (AOR = 1.31; 95% CI: 1.12–1.61) in children living in areas where distance to health facility is not a big problem compared to children living in areas where distance to health facility is a big problem (Table 4).

### Measures of variation (random-effects) and model fit statistics

As the results of multilevel logistic regression analysis depicted in Table 5, the null model (Model I) revealed statistically significant variation in childhood MCV1 vaccination across communities [**τ = 2.68,p < 0.001**], in which 45% variation in the odds of a child being MCV1 vaccinated is attributed to community-level factors (ICC =45%).

**Table 5:** Measures of variation (random intercept models) and model fit statistics in childhood MCV1 in Ethiopia, 2016

Measures of variation	Model 1^a^	Model 2^b^	Model 3^c^	Model 4^d^
Community level				
Variance (SE)	2.676 (0.091)*	0.556 (0.085)*	1.117 (0.074)*	0.479 (0.086)*
PCV (%)	Reference	79.2	58.3	82.1
ICC (%)	44.9	14.5	25.3	12.7
MOR^e^	4.73	2.03	2.73	1.93
Model fit statistics				
DIC (−2log likelihood)	4462	2984	4142	2953
AIC	4466	3050	4170	3043

*SE* Standard Error; *PCV* Proportional Change in Variance, *ICC* Intraclass Correlation Coefficient, *MOR* Median Odds Ratio, *DIC* Deviance Information Criterion, *AIC* Akaike’s Information Criterion

^a^Model 1 is an empty model, a baseline model without any explanatory variable

^b^Model 2 is adjusted for individual-level factors

^c^Model 3 is adjusted for community-level factors

^d^Model 4 is final model adjusted for both individual and community-level factors

^e^Increased risk (in median) that one would have if moving to a neighborhood/cluster with a higher risk

*P-value < 0.001

After adjusting the model for individual level factors (Model 2), the variation in the odds of a child receiving MCV1 remained statistically significant [***τ =*** **0.56*,p <*** **0.001**] across the communities, with 79% of variation in the odds of children who received MCV1 was attributed to the individual factors and 15% of the variance in MCV1 among the children was attributed to community-level factors.

Model 3, which is adjusted for community-level factors, revealed an increased variance of a child being vaccinated for MCV1 [***τ =*** **1.12*,p <*** **0.001**] across the communities, as compared to the variance reported in model 2. In this model, the community level factors explained the 58% of the variability in the odds of children receiving MCV1 (PCV = 58.3%), and 25.3% of the variation among the clusters was attributed to community level factors (ICC = 25.3%).

The final best-fit model (model IV), which adjusted for both individual and community-level factors simultaneously, depicted statistically significant variability to the odds of a child being MCV1 vaccinated [***τ =*** **0.48*,p <*** **0.001**]. In this final best fit model, about 13% of the variability among communities in the odds of a child being MCV1 vaccinated was due to the community-level factors (ICC = 12.7%) and about 82% of the variance in the odds of MCV1 vaccination (PCV = 82.1%) across communities was attributed to both individual and community-level factors.

In this study, the MOR shows the extent to which the child probability of receiving MCV1 is determined by residential area and is therefore appropriate for quantifying contextual phenomena. It quantifies the variation in MCV1 between clusters (the second-level variation) by comparing two children from two randomly chosen, different clusters. MOR greater than 1 in all models suggests a considerable between-cluster variation in childhood MCV1. Including both individual, and community level factors reduced the unexplained heterogeneity in MCV1 between communities from MOR of 4.73 in the null model to the MOR of 1.93 in the final model.

## Discussion

In this study, a total of 3722 children aged 12–35 months nested within 611 clusters were included from the 2016 EDHS data. The prevalence of childhood MCV1 was found to be 54.3% (95% CI = 52.7–55.9) which was low compared to the recent DHS reports of most low and middle income countries such as Egypt, 96% [31], Kenya, 87% [32], Rwanda, 95% [33], Ghana, 89% [34], Zimbabwe, 82% [35] and Uganda, 80% [36]. It was also low as compared to a local survey finding in the selected zones of Ethiopia by JSI-l10k, 80% [37], the national report in 2015, 87% [38]. In addition the prevalence of MCV1 is lower than the 2016 national target for childhood MCV1, 91% [38] and the recommended herd immunity threshold, 95% [8, 39]. This childhood MCV1 coverage below the recommended herd immunity threshold (95%) may indicate that not only the country but also the regions with high levels of childhood MCV1 coverage may still be at considerable risk for measles outbreaks.

Exploring spatial heterogeneity in childhood vaccination is gaining attention at all spatial scales to identify gaps and intervene accordingly [40]. In the global spatial autocorrelation analysis of this study, a clustering pattern of childhood MCV1 across the study areas was observed (Global Moran’s I = 0.134, *p*-value< 0.0001). This indicates that approximately the same coverages of childhood MCV1 were aggregated in specific areas. Hence, the SaTScan spatial analysis detected seven statistically significant most likely SaTScan clusters of areas with low MCV1 coverage. The most likely primary SaTScan cluster of areas with low MCV1 coverage was detected in Afar region; specifically in Zone 1, Zone 2 and Zone 4 administrative zones (LLR = 33.79, *p* < 0.01), and the most likely secondary SaTScan cluster in Somali region, specifically in Afder, Shabelle, Korah, Doolo, Nogob, Jarar and Fatan administrative zones (LLR = 26.76, *p* < 0.01). The 3rd and 4th most likely clusters of areas with low MCV1 coverage were detected in Gambella region; specifically in Nuer and Agnuak Administrative zones (LLR = 17.73, *P* < 0.01) and Afar region, particularly in Zone 2 administrative zone (LLR = 14.54, *P* < 0.01), respectively. In addition, the 5th most likely SaTScan cluster (LLR = 14.49, *P* < 0.01) was detected in Illuababur & Jimma Administrative zones of Oromia Region, while the 6th most likely SaTScan cluster (LLR = 14.18, *P* < 0.01) in zone 3 and zone 4 administrative zones of Afar region, North Shewa zone of Amhara region, West and East Hararge zones of Oromia region and Siti zone of Somali region. The last (7th) most likely SaTScan cluster (LLR = 12.27, *P* < 0.01) was detected in East Hararge zone of Oromia region, Fatan zone of Somali region, and Harari region. This local clustering of low childhood MCV1 coverages indicates that children who lived in the above mentioned geographical locations had a low probability of receiving MCV1 compared with those who lived outside the SaTScan clusters. It may be due to differences in health service accessibility and utilization, as well as socio-cultural differences in the community. This geographical clustering low MCV1 coverage may suggest that regions with high coverage of childhood MCV1 may be at risk for measles outbreaks [41].

As the multilevel analysis showed, the variation in the childhood MCV1 vaccination was attributed to both individual and community level factors. In the final model (model IV), individual and community-level factors accounted for about 82.1% of the variation observed for childhood MCV1. It is supported by other findings in Ethiopia [42] and Democratic Republic of Congo [43].

Children aged 24–35 months were more likely to receive MCV1 compared to children aged 12–23 months (AOR = 1.53; 95% CI: 1.25–1.88). This could be due to late initiation of childhood vaccination and Vaccine hesitancy for early ages. This indicates delaying in childhood vaccination which extends the period of vulnerability of children to vaccine preventable diseases [44]. We also found that children who have received the first dose of pentavalent vaccine (AOR = 9.09; 95% CI: 6.86–12.03) and third dose of pentavalent vaccine (AOR = 7.12; 95% CI: 5.51–9.18) were more likely to receive MCV1. This may be due to differences in childhood vaccination service availability and utilization among communities. It could also be justified as; women whose children lacked the first and third doses of pentavalent vaccination may miss the opportunities for information on the importance of childhood measles vaccination. Pentavalent first dose vaccination coverage is a good proxy indicator for the availability of access to and initial use of childhood vaccination services [13]. Hence, a high proportion of pentavalent first dose coverage may indicate the availability of childhood vaccination services in the communities. In addition, the third dose pentavalent vaccination coverage indicates the continuity of use by parents or care takers, client satisfaction with services, and capability of the system to deliver a series of vaccinations [13]. Hence, a high proportion of pentavalent third dose vaccination may indicate better childhood vaccination service utilization and the health system performance in relation to vaccination services.

Maternal education was identified as a strong predictor of childhood immunization in other several studies [45–48]. Children from mothers with secondary or higher education had higher odds of being vaccinated for MCV1 (AOR = 1.62; 95% CI: 1.03–2.55). This finding is supported by other studies in Ethiopia [42], Democratic Republic of Congo [43], Nigeria [49, 50], Kenya [51] and in India [52] and it is not surprising that educated mothers are generally more likely to utilize health care services including childhood immunization services, and have better communication skills [53, 54].

From the community level characteristics, geographical region was a significant predictor of childhood MCV1. Children who were living in Afar, Oromia, Somali, Gambella and Harari regions were less likely to receive MCV1 as compared to children lived in Addis Ababa. This could be justified by the regional differences in some background characteristics such as culture, religion, economical status, vaccine supply, and availability and accessibility of immunization health services.

In contrast to the findings that have been documented elsewhere [55–58], maternal place of residence was not a significant predictor of childhood MCV1. This may be due to differences in study period, and sample size. However, the finding is consistent with the findings of other studies conducted in central Ethiopia and Nigeria, in which a non-significant association between place of residence and likelihood of childhood MCV1 [45, 59]. This may suggest a need for further investigation.

Finally, this study revealed that children who lived in areas where distance from health facility is not a big problem were more likely to receive childhood MCV1 that is consistent with a study finding in sub-Saharan Africa [60].

EDHS is a national and subnational representative household survey with a high response rate and the findings are generalizable to the national and subnational populations. Hence, generalizability is the strength of this study. Applying spatial scan statistics and employing multilevel regression models to identify both individual and community level factors that could not be addressed with ordinary logistic regression model are other important strengths. However, recall bias may be introduced due to the retrospective nature of the data, and the maternal verbal reports for childhood vaccination.

## Conclusion

A clustered pattern of areas with low childhood MCV1 coverage was observed in Ethiopia. Statistically significant local clusters of areas with low childhood MCV1 were detected in Somali, Afar, Gambella, and Oromia regions of the country. Both the individual level characteristics (child’s age, first and third doses of pentavalent vaccination, maternal age and education) and community level characteristics (geographic region and distance from health facility) were statistically significant predictors of childhood MCV1. Hence, it is good if the federal ministry of health and other concerned child health programmers give priority of the areas with low MCV1 coverage identified in this study. It is also better to consider the individual and community level determinant factors identified in this study.

## Data Availability

The datasets generated and/or analyzed during the current study are available in the MeasureDHS Program repository, (https://www.dhsprogram.com/data/dataset_admin) to all registered users.

## Abbreviations

AIC: Akaike’s information criterion
AOR: Adjusted odds ratio
CI: Confidence intervals
COR: Crude odd ratio
DIC: Deviance information criterion
DPT-HepB-Hib: A combination vaccine containing diphtheria, pertussis, tetanus toxoid, hepatitis B and Haemophilus influenza type b
EAs: Enumeration areas
EDHS: Ethiopian demographic and health survey
GIS: Geographic information system
ICC: Intra-cluster correlation
IR: Incidence rate
LLR: Log likelihood ratio
MCV1: First dose of measles-containing vaccine
MOR: Median odds ratio
PCV: Proportional change in variance
SD: Standard deviation
SNNPR: Southern Nations, Nationalities, and People Region
WHO: World health organization
